# Using a global framework for health workforce development: National case studies on continuing professional development in pharmacy

**DOI:** 10.15694/mep.2019.000044.1

**Published:** 2019-03-11

**Authors:** Lina Bader, Banan Mukhalalati, Ahmed Awaisu, Toyin Tofade, Ian Bates

**Affiliations:** 1 International Pharmaceutical Federation; 2 Qatar University; 3 Howard University; 4 UCL School of Pharmacy

**Keywords:** Health workforce, Pharmacy, health, workforce development, continuing professional development, CPD, CE, Pharmaceutical Workforce Development Goals, workforce transformation, education & training

## Abstract

This article was migrated. The article was marked as recommended.

Investing in the education, training and development of the global health workforce is integral to achieving global health goals, including universal health coverage and access to safe and quality services. Systemising and formalising CPD & CE for health workers as part of their professional development has become a central component of health workforce development. A global framework for pharmacy workforce development support countries to assess their CPD & CE development needs and facilitate progress towards global goals. This paper describes this global framework and how it’s being used and implemented in countries worldwide.

## Introduction

Continuing professional development (CPD) is now a mandatory activity for most regulated health professions. Investment in the capacity, education and training of the global health workforce is key to improving global health goals and access to safe and effective medicines and health services ( Wheeler and Chisholm-Burns, 2018; International Pharmaceutical Federation (FIP), 2019; FIP Council, 2002). As with other professions, many countries have begun to use CPD, as opposed to more formal continuing education (CE), for prelicensure, license renewal and competency development of pharmacy professionals. The main benefit of CPD is to provide the practitioner with a systemised way to learn and to develop individual competencies as well as to assist with practice and health system gaps ( Wheeler and Chisholm-Burns, 2018). The process of CPD should ideally instil a practice of self-directed, lifelong learning in the user.

The importance of developing CPD and CE as a means to lifelong learning in pharmacy has long been recognised as a priority area for advancement by the global pharmacy leadership body. However, there is variation around the world and not all countries use CPD to the same extent, or with equity in weighting and importance. This paper aims to describe the development of CPD/CE in the pharmacy profession and the global roadmap set for it by the International Pharmaceutical Federation (FIP), as well as to discuss selected national case studies on CPD in pharmacy using the framework of the globally adopted Pharmaceutical Workforce Development Goals (PWDGs). This ultimately highlights progress and trend, but importantly, the global needs and drivers for development.

## CPD and CE in the pharmacy workforce: Background and context

FIP is the global leadership body representing pharmacy and pharmaceutical sciences. Through its 144 national organisations, academic institutional members and individual members, FIP represents over four million pharmacists and pharmaceutical scientists around the world ( International Pharmaceutical Federation (FIP), 2019). Since its establishment in 1912, FIP has worked to support the development of the of professional standards, which expect pharmacists to observe in discharging their professional responsibilities. Within the context of CPD, FIP published a Statement of Professional Standards ( FIP Council, 2002) which states that: “maintaining competence throughout a career, during which new and challenging professional responsibilities will be encountered, is a fundamental ethical requirement for all health professionals”. In the Statement, a distinction between CPD and CE has also been made in which CPD is defined as “the responsibility of individual pharmacists for systematic maintenance, development and broadening of knowledge, skills and attitudes, to ensure continuing competence as a professional, throughout their careers.” While CE is an important component of CPD, CPD is more than participation in Continuing Education (CE) which, on its own, does not necessarily lead to positive changes in professional practice nor does it necessarily improve healthcare outcomes ( Davis *et al.*, 1999). FIP has begun the process of revising the Statement to reflect consensus from experts on CPD around the world through the FIP CPD/CE working group.

In 2014, the FIP Global Report on CPD/CE was published ( International Pharmaceutical Federation (FIP), 2014), which summarized and described the literature, data and frameworks of CPD around the world. The intention of the report was to assist countries in identifying the scope of CPD/CE that they were willing to endorse in their region. The report provided a global description of CPD/CE in pharmacy, which featured key messages on CPD. The report also discussed the results of a survey deployed to collect data on providers of CPD, funding sources, regulatory and licensing requirements, maintenance requirements, and development activities for pharmacists and technicians. The report also showcased selected country CPD frameworks for the purposes of sharing how diverse the world is with respect to CPD frameworks. Nine country case studies were featured in the report (Australia, Canada, Croatia, Japan, Namibia, New Zealand, Northern Ireland, Oman, UK, and United States of America). Each country described their current drivers, implementation challenges, lessons learned, key tools that helped in each CPD stage, national strategies for health care services and future plans. Lastly, a description of how CPD was linked to other education activities in FIP was provided. In summary the emphasis on life long learning skills was recognised as paramount to the implementation of a robust CPD/CE process. Pharmacy professionals are encouraged to utilize the tool to obtain specific data, and to guide future plans in their organization, community or country. Ultimately, the first step is identifying gaps that may exist in learning. The FIP competency framework is a good first place to begin in identifying that for all pharmacy professionals.

The report offered additional insight into how CPD is organised and systemised in some countries using case examples. For example, the use of the cyclical approach, unique to CPD, comprising of *reflect*, *plan*, *act* and *evaluate* has been in used in Canada for some time now specifically in Ontario and Quebec and as a hybrid system in Alberta and British Columbia. CPD in pharmacy is compulsory in Namibia, though there remain challenges with capacity and resources. Japan’s national pharmaceutical association introduced the system in 2009 while New Zealand’s pharmacy council endorsed the CPD framework in 2005. In Northern Ireland the department of Health funded the development of a system of CPD for pharmacists in 2004 and the Oman Ministry of Health has also put in place systems to support pharmacists CPD. The report also describes how the UK has implemented a system of CPD for preceptors and faculty to ensure ongoing lifelong learning. In 2006, the US began the process of implementing the CPD process by conducting a pilot study which involved training five state representatives to enrol participants into a placebo group and a CPD process group. Since then the CPD process has been included in the accreditation standards of the PharmD program and is an expectation for faculty students and staff in academia.

## No workforce development without CPD/CE

The Nanjing Conference on Pharmacy and Pharmaceutical Sciences Education was convened in 2016 in Nanjing, China. Several representatives from around the world came together to adopt a global workforce roadmap that would influence pharmacy and pharmaceutical sciences education and workforce development worldwide. The roadmap is represented in three milestone documents: (1) the Global Vision for Education and Workforce ( International Pharmaceutical Federation (FIP), 2016a); (2) the Pharmaceutical Workforce Development Goals (PWDGs) ( International Pharmaceutical Federation (FIP), 2016c); and (3) the Statements on Pharmacy and Pharmaceutical Sciences Education “Nanjing Statements” ( International Pharmaceutical Federation (FIP), 2016b).

The PWDGs are a set of 13 measurable, feasible and achievable goals that progress the achievement of the Global Vision. Together with the Nanjing Statements, the roadmap addresses the development of the workforce from initial education to advanced practice. The PWDGs are divided into 3 clusters: Academy, Professional Development, and Systems. Within the Systems cluster, PWDG “9” “CPD Strategies” call for all countries and territories to have all professional development activities clearly linked with needs-based health policy initiatives and pharmaceutical career development pathways. Some of the indicators for this goal include: evidence of an effective CPD strategy according to national and local needs; development of programmes to support professional development across all settings of practice and all stages of a pharmacist’s career - ideally, this should be linked with all professional development activities across the workforce; and education in CPD strategies and self-directed behaviours should be initiated at the student level.

The Nanjing Statements are a set of 67 statements that describe the envisioned future for pharmaceutical education needed to enhance professional standards worldwide and are intended for education providers, including schools of pharmacy and CPD/CE providers. The Statements comprise eight focus areas referred to as “clusters” and these are: shared global vision; professional skills mix; recruitment of students; foundation training and leadership; experiential education; resources and academic staff; quality assurance; and continuing professional development (CPD). The CPD Cluster has four statements presented and endorsed by the delegates. These were:

1. CPD should apply both to those in the regulated professional practice and to those working in unregulated professional practice, such as academia and the pharmaceutical sciences.
2. All members of the pharmaceutical workforce should accept a responsibility to manage their own CPD.
3. Promotion of CPD should begin with students at the start of their education.
4. Schools and faculties should support CPD for graduated professionals to prepare them for advanced practice roles.

### Current status and progress of CPD in pharmacy: Case study in Qatar

In 2017, a global survey on pharmacy workforce development analysed progress on PWDG “9” across 14 countries as part of a wider study on global progress against the PWDGs ( International Pharmaceutical Federation (FIP), 2017). The results, consistent with the FIP 2014 synthesis of 9 country case studies, report a wide variation in the way CPD is systemised, regulated and delivered around the world. Portugal’s Pharmaceutical Society oversees a CPD credit system that awards pharmacists’ educational and professional activities with a numeric value. Credits attributed consider hours of a number of activities including: learning, practice, publication, tutoring/mentoring pre-registration students and patient monitoring, to name a few. Similarly, in Malaysia the pharmacy workforce needs to collect a minimum set of credit hours to retain their professional license. Linking CPD to recertification is a common trend worldwide; for example, Costa Rica’s professional association has developed a functional Pharmaceutical Professional Recertification System which is linked to maintaining CPD and Singapore has mandated CPD for all pharmacists. A number of countries still report an absent or less than optimal CPD system that requires further development. For example, South Africa reported that there is no national CPD strategy in the country, but that the introduction of the new competency standards in South Africa will be a driver for the development of a CPD system. Similarly, having a future national CPD strategic plan is reported to be needed in Zambia.

In Qatar, a recent separate study was conducted in 2018 to examine the alignment of pharmacy practice and pharmacy education with the FIP’s PWDGs in the country. A conventional Delphi method utilizing a validated self-assessment survey tool developed by the FIP was used to collect data from a panel of experts in the College of Pharmacy at Qatar University and the Qatar’s Ministry of Public Health. Content analysis was used to analyse data, prioritize the identified gaps, and to provide recommendations and solutions to address these gaps. Data about each indicator representing the PWDG “9”, CPD strategies goal, were individually analysed, in order to understand the current status of this goal.

The first indicator focuses on evidence of an effective CPD strategy according to national and local needs. In Qatar, CPD activities are designed with consideration to national needs and health-related problems such as cancer, diabetes, and cardiovascular diseases. Nationally-accredited providers of CPD need to demonstrate the evidence of utilization of tools (minimum of two) to assess the needs of CPD programs’ participants. The second indicator centres on the development of programs to support professional development across all settings of practice and all stages of a pharmacist’s career. In Qatar, there is currently no professional pharmacy organization to facilitate and promote CPD programs and activities across all practice settings. Furthermore, stakeholders reported that there is a lack of developmental frameworks describing professional competencies and scope of practice for all stages of professional careers, which is PWDG “5”. As a result, CPD activities do not take into account the different stages of pharmacists’ career, because these stages are not yet clearly defined.

The absence of a pharmaceutical professional leadership body in Qatar and the lack pharmacy practice developmental frameworks may negatively impact the application of PWDG 9’s third indicator, which emphasizes on the availability of programs or bodies that link all professional development activities across the workforce and ensures the development of pharmacists at different stages of their career. The fourth and last PWDG “9”’s indicator stresses that education in CPD strategies and self-directed learning behaviours should be initiated at the student level. In that regard, undergraduate and graduate students in the College of Pharmacy at Qatar University, which is the only college of pharmacy in Qatar, are encouraged and sponsored to attend and actively participate in CPD activities and conferences nationally and internationally. This participation helps to develop students professionally, and to enhance their lifelong and self-directed learning skills and behaviours. For practicing pharmacists, self-directed learning is rewarded by earning CPD points, which are mandatory for licensing renewal.

### Achieving PWDG “9”: Implementation of all inter-related PWDGs

It is therefore clear from the discussed national case studies that the globally adopted PWDG “9”, CPD Strategies, cannot be achieved in isolation, and that effective CPD development begins at early education. Achieving PWDG “9” is therefore closely linked to and dependent on progressing at the three PWDGs in the Academy cluster. Firstly, PWDG “9” is dependent on Academic Capacity (PWDG “1”), which focuses on ensuring that educational institutions are providing their students with the skills they need to be reflective, self-directed learners; the Nanjing Statements can be used as a tool to assess this capacity.

Similarly, achieving PWDG “9” is connected to Foundation Training Systems, (PWDG “2”), which emphasizes incorporating CPD elements as part of their assessment. Finally, PWDG “9” is linked to Quality Assurance of Education (PWDG “3”), which facilitates the incorporation of CPD/CE elements in education by setting higher education standards. Other interactions with PWDG “9” include: Advanced and Specialist Practice Development (PWDG “4”) Competency Development (PWDG “5”), and Workforce Policy Formation (PWDG “13”).

## Conclusions

Despite having a more or less common global definition to pharmaceutical CPD, there is variation in the mandated weighting and in the scope and practice of this developmental method; processes may differ as does extent. Few countries are using CPD as a competency-development method, with many more using CPD as an “update” process for continued knowledge-based learning, and others as a means to recertification.

The role of a national professional association in determining a clear national workforce vision that engages all stakeholders is key to progressing CPD Strategies, PWDG “9”. The recent study in Qatar provides a model for conducting a comprehensive needs-assessment using the PWDGs to determine gaps in progress, which will in turn identify the priority areas for development on a national level.

Having nationally-identified professional leadership for CPD is a clear prerequisite for impactful workforce development; global leadership can add to the national leadership by making available systematic frameworks for workforce development. The FIP “Nanjing Outcomes” are the first time that CPD can be aligned with other essential criteria for workforce transformation.

With an escalating global health crisis, and WHO estimate a shortfall of around 18 million health workers by 2030, a national professional CPD becomes an essential ingredient in health workforce construction. Monitoring and supporting national reform and transformation of CPD has become critical for a fully functional healthcare workforce.

## Take Home Messages

A global framework for health workforce development, the Pharmaceutical Workforce Development Goals, offers countries and other professional associations a roadmap for progressing CPD & CE.

## Notes On Contributors

Dr. Lina R. Bader, Lead for Workforce Transformation & Development, International Pharmaceutical Organisation, The Hague, Netherlands.

Dr. Banan Mukhalalati, Assistant Professor, College of Pharmacy, Qatar University.

Dr. Ahmed Awaisu, Associate Professor, College of Pharmacy, Qatar University.

Dr. Toyin Tofade, Dean and Professor, College of Pharmacy, Howard University, 2300 4 ^th^ Street NW, Washington DC, USA.

Prof. Ian Bates, Director of FIP-UCL CC, School of Pharmacy, University College London, London, UK.
