# Supplementary material for: Using a global framework for health workforce development: National case studies on continuing professional development in pharmacy
Source: MedEdPublish (2016). 2019 Mar 11;8:44. [Version 1] doi: 10.15694/mep.2019.000044.1 (PMC10712579; doi:10.15694/mep.2019.000044.1)
Supplement: Peer review history

# Reviewer report: Reviewer response for version 1

**DOI:** 10.21956/mep.19798.r29778

**Published:** 2019-09-06

**Authors:** Megan Anakin

**Affiliations:** 1 University of Otago

This review has been migrated. The reviewer awarded 2 stars out of 5 This article will be of interest to pharmacy educators with a strong interest in workforce development. The authors partially meet the two goals stated in the abstract, i.e. to describe:• a global framework for pharmacy workforce development • how the framework is being used and implemented in countries worldwideThe focus on CPD and CE, with an international pharmacy audience in mind, is a key strength of the paper. The authors note that CPD and CE for pharmacists vary globally in their use, equity in weighting, and importance. They also describe the work FIP is doing to clarify the definitions of CPD and CE, and report on this activity from a global perspective. A benefit of this approach allows for CPD/CE processes to be compared and for pharmaceutical workforce development goals to be generated from this information. Currently, FIP has identified 13 goals to guide CPD/CE practice. A case study in Qatar is used to illustrate how progress can measured in relation to the goals. The paper would benefit from a list of definitions for the 13 goals so readers can more easily navigate the discussion of the case study of Qatar. In addition, to enhance this article, the authors might consider expanding the use of references to the wider literature about CPD/CE to help the reader understand the significance of this group’s efforts to improve the pharmacy workforce globally and make connections of workforce development in other health professions. These connections are made at the beginning of the article but are lacking later on. Ideas about how this material might be relevant to practicing pharmacists and pharmacy educators would also enhance this article. Implications of this approach to fostering CPD/CE workforce development other health professions would capture the interest of a wider audience.

Reviewer Expertise:

NA

No decision status is available

# Reviewer report: Reviewer response for version 1

**DOI:** 10.21956/mep.19798.r29777

**Published:** 2019-04-13

**Authors:** Hossam Hamdy

**Affiliations:** 1 Gulf Medical University

This review has been migrated. The reviewer awarded 3 stars out of 5 This case study attracts the attention to the importance of continuing professional development and life long learning for pharmacists. It is well known that Health Professions Education is inseparable from the Healthcare System of the country. It is context related. The introduction and aim of the case study are clear. Method: The authors description of the methodology used in studying the situation in Qatar needs to be expanded. The questionnaire used for collecting data could be an attachment to the paper while the key questions should be mentioned in the text. How many participated in the Delphi exercise? Are they only from the College of Pharmacy? What about other stakeholders? Results and discussion of the finding in Qatar should be the main focus of the case study. The lessons learned from the findings, are they going to the applied, By whom? How other countries can benefit from the results in Qatar? All the references are related to Pharmacy except one 20 years old!!The Excellent Systemic review published by Yvonne Steinert titled ‘A Systemic review of faculty development initiatives designed to improved teaching effectiveness in medical education” should be read and included. This study is important. CPD in pharmacy education can benefit from it. As it is, I consider it acceptable. If revised it can be definitely of a higher value.

Reviewer Expertise:

NA

No decision status is available

# Reviewer report: Reviewer response for version 1

**DOI:** 10.21956/mep.19798.r29779

**Published:** 2019-03-25

**Authors:** Trevor Gibbs

**Affiliations:** 1 AMEE

This review has been migrated. The reviewer awarded 2 stars out of 5 Thank you for asking me to review this paper. As the authors point out the topic of CPD is very important for all health professionals, so I was looking forward to reading this paper which suggested that it would describe a global framework for development. I subsequently found this paper very difficult to read and understand and after reading I was still looking for this Global Framework. I thought that the paper lacked structure and flow and its take home message failed to connect to the paper

Reviewer Expertise:

NA

No decision status is available

# Reviewer report: Reviewer response for version 1

**DOI:** 10.21956/mep.19798.r29776

**Published:** 2019-03-12

**Authors:** Ken Masters

**Affiliations:** 1 Sultan Qaboos University

This review has been migrated. The reviewer awarded 2 stars out of 5 The paper gives an overview of some aspects of CPD in Pharmacy. While the paper is an interesting read, it tends to be a collection of short pieces rather than a collective whole. As a result, there is somewhat of a disjuncture between the title, the abstract, and the paper itself. That does not mean that the paper has no value, but I really do think that the authors have some work to do on it.• The title indicates that the paper will present “case studies on continuing professional development in pharmacy”. The abstract, however, focuses on the framework, and only the last line refers (without using the term “case studies”) to the issue of looking at the framework in different countries. So, some adjustment of the title or the abstract is required.• After the Introduction, the next large section (“CPD and CE in the pharmacy workforce: Background and context”) deals with the report. Although case studies in the report are mentioned, the focus is on the report. This is a report on a report. • Similarly, the next part of the paper (“No workforce development without CPD/CE”) deals with the results of the Nanjing Conference; again, however, no case studies are presented; this is a conference report.• The third section of the paper (“Current status and progress of CPD in pharmacy: Case study in Qatar”) implies that is will be a case study of Qatar. Most of that section, however, discusses a survey (in which brief mention of some countries is given). The Case Study part is confined to a very small section of the paper: 3 paragraphs, taking up about ¾ of a page.• The final section of the paper, before the Conclusion, reflects on the PWDG "9", and refers to the previous case studies, but only one case study (on Qatar) has been presented.• The Take Home message does not even mention the case study.So, we have a paper that has been classified as “Case study”, that promises “case studies” in the title, but delivers only one Case Study, taking up less than 20% of the paper (excluding references). I would recommend that the authors decide on one of two possible paths when re-submitting version 2 of the paper:• Either it should be presented as a case study of Qatar, in which case the other material should be substantially shortened, and the discussion of Qatar substantially lengthened, or• It is presented as a case study of two or three countries (making it “case studies”), in which case, the other material should still be shortened, but the other two countries should receive approximately the same amount of space as Qatar. In this case, the Abstract, Introduction, Conclusion and Take Home message should more accurately reflect the changes.By correcting these errors, the focus and aim of the paper will be clearer, and then the value of the paper can be properly assessed.Some other points:• Abbreviations (such as CPD and CE) should be written out in full, before being used as abbreviations, including in the Abstract.• There are several errors is this sentence that need correcting: “…development of the of professional standards, which expect pharmacists…”

Reviewer Expertise:

NA

No decision status is available

# Reviewer report: Reviewer response for version 1

**DOI:** 10.21956/mep.19798.r29775

**Published:** 2019-03-11

**Authors:** P Ravi Shankar

**Affiliations:** 1 American International Medical University

This review has been migrated. The reviewer awarded 4 stars out of 5 Thank you for inviting me to review this interesting manuscript. The authors provide an overview of continuing professional development (CPD) and continuing education (CE) in Pharmacy. They provide a global framework of CPD in pharmacy and also describe a few country specific case studies. The major emphasis of the discussion is on the pharmaceutical workforce development goals (PWDGs). The authors mention that the PWDGs are set of 13 measurable goals and then focus on the 9 goals in the systems cluster (PWDG9). The PWDG 9 can be described in greater detail. The authors also mention the Nanjing statements. Listing the PWDG9 and better explaining the link between the Nanjing statements and the PWDG9 will be useful to help readers better understand the paper. Some of this could be achieved through the use of tables. The manuscript will be of special interests to all pharmacy educators.

Reviewer Expertise:

NA

No decision status is available
